# Which is preferred for initial treatment of papillary thyroid cancer, total thyroidectomy or lobotomy?

**DOI:** 10.1002/cam4.3743

**Published:** 2021-01-29

**Authors:** Zhen Wu, Lin Han, Wenlei Li, Wei Wang, Liqaing Chen, Yumin Yao, Yongkun Wang

**Affiliations:** ^1^ Department of Thyroid Surgery Liaocheng People's Hospital Clinical Hospital of Shandong First Medical University Liaocheng China; ^2^ Department of Pathology Liaocheng People's Hospital Clinical Hospital of Shandong First Medical University Liaocheng China

**Keywords:** initial treatment, metastasis, surgery, thyroid cancer

## Abstract

**Purposes:**

The incidence of thyroid cancer has increased annually, and has a heavy psychological and economic burden on society and individuals. Based thyroid cancer data from patients treated in Liaocheng People's Hospital from 2015 to 2018, with Chinese national and regional characteristics, in this study, we addressed the controversy of which initial thyroid surgical mode, lobectomy or total thyroidectomy, is most effective.

**Methods:**

Clinical and pathological data from 2108 patients with thyroid cancer, who were initially diagnosed and treated surgically, were collected from the Department of Thyroid Surgery. Among them, there were 1001 cases who underwent open operation with total thyroidectomy + central lymph node dissection; meanwhile, 1107 cases were treated with neck lateral lymph node dissection at the same time.

**Results:**

The overall metastasis rate of all patients was 57.23%. Even the lymph node metastasis of papillary thyroid microcarcinoma (PTMC) was as high as 48.97%. When the mass rose above 2 cm, the proportion of metastasis increased to 77.22%. When the tumor was complicated with bilateral and multiple high‐risk factors, the proportion of metastasis was 65.27% and 72.21%, respectively. When the tumor breaks through the capsule, the metastasis rate was 67.08%. With the increase of tumor diameter, the metastasis of cervical lymph nodes ranged from 22.54% to 73.33%, which showed positive correlation. 49.32% of patients had lymph node metastasis in the lateral cervical region. When the diameter of the tumor reached T1c level, the metastasis of the cervical lymph nodes was 56.91%, and the number of metastatic cases above T1c level accounted for 69.96% of the total metastatic cases.

**Conclusion:**

The degree of malignancy of thyroid cancer depends on tumor genome evolution. Rates of neck lymph node metastasis are high, particularly among patients with risk factors for poor prognosis. It is recommended that initial treatment should comprise at least total thyroidectomy + central lymph node dissection in China, to avoid the risks associated with secondary surgery and effects on patient quality of life. When the tumor diameter exceeds 1 cm, the risk of cervical lymph node metastasis is high, and we recommended lateral lymph node dissection.

## Graphical abstract

There were 1001 cases who underwent open operation with total thyroidectomy + central lymph node dissection, meanwhile 1107 cases were treated with neck lateral lymph node dissection at the same time. It is recommended that initial treatment should comprise at least total thyroidectomy + central lymph node dissection in China, to avoid the risks associated with secondary surgery and effects on patient quality of life. When the tumor diameter exceeds 1 cm, the risk of cervical lymph node metastasis is high, and we recommended the lateral lymph node dissection.

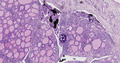

## 1 INTRODUCTION

In the past 30 years, the incidence of thyroid cancer has been increasing annually worldwide, with the incidence in China also rising. In 2012, the number of new cases and deaths from thyroid cancer in China accounted for 15.6% and 13.8%, respectively, of the global total. It is estimated that by 2019, thyroid cancer will become the third most common malignancy in women in the United States, leading to medical expenditure of approximately $19–21 billion, representing a heavy economic burden on both society and individuals.^1^

Differentiated thyroid carcinoma accounts for 80%–90% of thyroid cancer incidence and the most common metastasis is the cervical lymph nodes, which seriously influence patient prognosis. Therefore, effective and appropriate surgical treatment of differentiated thyroid carcinoma is of great clinical significance. Total thyroidectomy and lobectomy remain the main primary surgical interventions for thyroid carcinoma. To date, there has been no large sample randomized controlled clinical trial to compare these two methods, hence their relative efficacy remains controversial and comparative studies have relied mainly on retrospective analyses.^2^ This indicates that, although the progress of papillary thyroid carcinoma (PTC) is slow, the ability and tendency to metastasize to lymph nodes and even distant organs are consistent with other malignancies. In general, it is unscientific to classify malignant tumors based on tumor size. The invasiveness and distant metastatic ability of tumors are derived from the evolution of the tumor genome.^3^, ^4^ There are still different opinions on whether to perform preventive lateral neck lymph node dissection. The focus of debate is on the following aspects: preventive lateral neck lymph node dissection needs to expand neck incision and affect appearance; lateral neck lymph node dissection can cause shoulder syndrome, which seriously affects the patient's quality of life; differentiated thyroid cancer is slow to develop, as long as it is closely followed by diagnosis, allowing treatment when suspicious lymph nodes are found.^5^, ^6^

We collected clinical and pathological data from 2108 patients undergoing treatment at the Department of Thyroid Surgery, Liaocheng People's Hospital, and analyzed their responses to current treatment approaches. Using accurate clinical data, we aimed to settle the controversy about appropriate treatments for thyroid cancer and determine the most appropriate initial surgical intervention for patients in China with this condition.

## 2 METHODS

This study was a retrospective analysis of a total of 2108 cases of PTC treated and undergoing surgery from 1 January to 31 December 2018 in the Department of Thyroid Surgery, Liaocheng People's Hospital, Clinical Hospital of Shandong First Medical University, China. All patients underwent fine needle aspiration before surgery, and had pathological diagnoses of papillary carcinoma or suspected papillary carcinoma.

Surgical procedures were as follows: all the enrolled patients underwent total thyroidectomy, and were randomly enrolled in the two surgical methods. 1001 cases were treated with total thyroidectomy + central node dissection. Total thyroidectomy + central lymph node dissection + lateral neck node dissection were performed in 1107 cases. For patients <18 years old, informed consent was obtained from both parents.

Surgical methods were based on the guidelines of Chinese Thyroid Cancer (CTA), following the experience of clinical professors. We confirm that all methods were performed in accordance with the relevant guidelines.

Written informed consent for participation in this study was obtained from each patient after full explanation of the purpose and nature of all procedures used. All patients agreed to receive total thyroidectomy. The study was conducted in accordance with the Declaration of Helsinki. The ethical approval number is 2016071.

Before surgery, patients and their families were fully informed and discussed the implications.

Pathological data were obtained from the Department of Pathology, Liaocheng People's Hospital, Clinical Hospital of Shandong First Medical University, and were based on postoperative analysis of paraffin‐embedded tumor samples. Pathological data were stored in a unified database, and comprise standard information, including preoperative fine needle biopsy cytology pathology, intraoperative rapid frozen pathology, and postoperative routine paraffin pathology. Information on all patients was systematically coded in a single computer file. Data from this system were exported for statistical analyses.

## 3 RESULTS

All patients were treated directly after discovery of the lesion, without any observation period. Cases included 416 males and 1692 females, and were 13–79 years old, with 285 cases aged ≤35 years (Table 1).

**TABLE 1:** The age and gender of patients

Age	Male	Female	Total
≤20	8	26	34
≤30	37	60	97
≤40	112	454	566
≤50	108	511	619
≤60	87	402	489
≤70	49	204	253
>71	15	35	50

**FIGURE 1:**
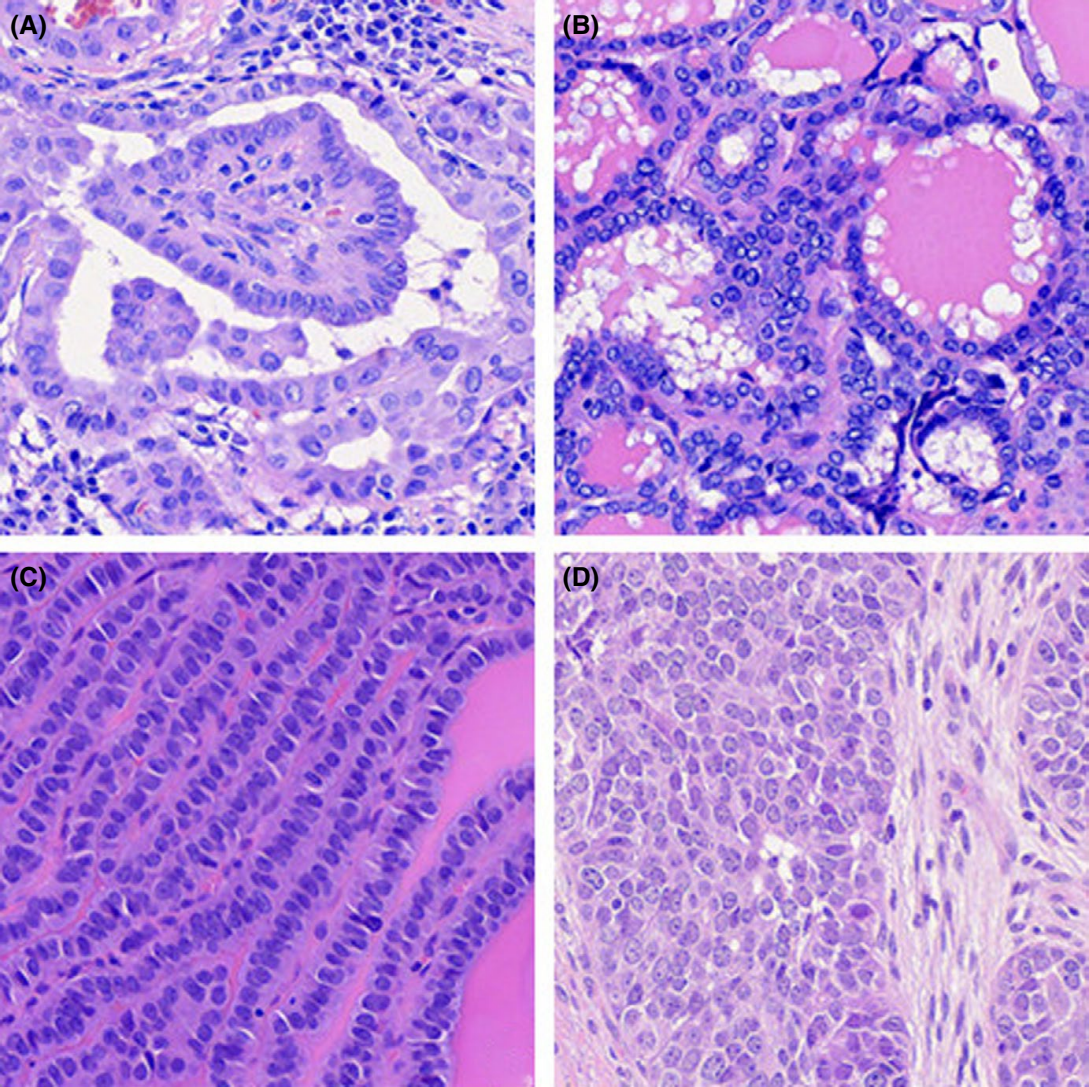
Histology types of thyroid cancer identified in this study. (A) Classical type papillary thyroid carcinoma (PTC). (B) Follicular variant PTC. (C) Tall cell variant PTC. (D) Solid variant PTC. All images are 40× magnification

The pathological subtypes present among the 2108 cases of PTC were classical (*n* = 2051), follicular variant (*n* = 45), tall cell variant (*n* = 8), and solid variant (*n* = 4) (Figure 1; Table 2).

**TABLE 2:** Thyroid cancer pathology, diameter, and metastasis

Pathological subtypes	Classical	Follicular variant	Tall cell variant	Solid variant
Number of cases	2051	45	8	4
Item	Case	Ratio	Metastasis	Ratio
T1a	442	20.97%	160	36.20%
T1b	720	34.16%	409	56.81%
T1c	604	28.65%	428	70.86%
T2	316	14.99%	244	77.22%
T3	26	1.23%	17	65.38%
Collection	2108		1258	57.23%

There were 2108 cases of thyroid papillary carcinoma, 1001 cases of total thyroidectomy + central lymph node dissection, and 1107 cases of total thyroid dissection + central lymph node dissection + cervical lymph node dissection. The central region of the lymph nodes was known as level Ⅵ, and the lateral cervical region was dissected in level Ⅱ–Ⅴ. The average metastasis rate was 57.23%. The proportion of metastasis was positively correlated with tumor diameter, from 36.22% to 77.22%. However, T3 patients may have a low rate of lymph node clearance due to irregular surgical treatment or intraoperative surgery strategies, resulting in deviations in data (Tables 2 and 3). The proportion of thyroid micropapillary carcinoma was 55.12%, and the lymph node metastasis rate was 48.97% (Table 4). According to our clinical practice, and to facilitate the statistics of clinical data, we artificially classified the tumor diameter as follows: T1a ≤ 0.5 cm, 0.5 cm <T1b ≤ 1 cm, 1 cm < T1c ≤ 2 cm, 2 cm < T2 ≤ 5 cm, T3 > 5 cm.

**TABLE 3:** The diameter and metastasis of thyroidectomy + central node dissection

Item	Case	Ratio	Metastasis	Ratio
T1a	300	29.97%	99	33.00%
T1b	370	36.96%	170	45.95%
T1c	235	23.48%	119	50.64%
T2	85	8.49%	41	48.24%
T3	11	1.10%	5	45.45%
Collection	1001		434	43.36%

**TABLE 4:** The proportion and clinical characteristics of patients with papillary thyroid microcarcinoma

Cases	Proportion	Metastasis	Proportion	Central lymph node	Proportion	Lateral neck lymph node	Proportion
1162	55.12%	569	48.97%	405	34.85%	164	14.11%

There were 773 bilateral tumors, accounting for 35.17%, and the transfer rate was 65.27%. There were 547 cases of multiple tumors, accounting for 24.89%, and the transfer rate was 72.21%, and 343 cases of bilateral combined multiple tumors, accounting for 15.61%, and the transfer rate was 74.05%. There were 483 cases of tumor broke through the membrane, accounting for 22.91%, and the metastasis rate was 67.08% (Table 5). When the tumor is combined with bilateral, multiple, and breakthrough membranes, the metastasis rate is higher than the average transfer rate, and the analysis has statistical significance.

**TABLE 5:** The surgery and high‐risk factors of thyroid cancer

Item	Case	Ratio	Metastasis	Ratio
Bilateral	773	35.17%	508	65.27%
Multiple	547	24.89%	395	72.21%
Both	343	15.61%	254	74.05%
Capsule invasion	483	22.91%	324	67.08%

In 1001 cases, only central lymph node clearance was performed, accounting for 47.48%, and the transfer rate was 41.86%. In 1107 patients, cervical lymph nodes were increased, accounting for 52.51%, and the transfer rate was 74.44% (Table 6). With the increase in tumor diameter, the overall metastasis rate increased from 42.96% to 87.88%, of which patients with T3 may have a relationship with surgical treatment or surgical strategy during surgery, and the lymph node metastasis rate (80%) is lower than that of T2 (87.88%), and there is a discrepancy in the data (Table 7). The metastasis rate of cervical lymph nodes was also positively correlated with tumor diameter, from 22.54% to 73.33%. Neck lateral metastasis occurred in 546 of all tumors, accounting for 49.32% (Table 7).

**TABLE 6:** The metastasis of thyroid cancer

Item	Case	Ratio	Metastasis	Ratio
Total thyroidectomy Central lymph node dissection	1001	47.48%	419	41.86%
Total thyroidectomy Central lymph node dissection Lateral neck node dissection	1107	52.51%	824	74.44%

**TABLE 7:** The metastasis of lateral neck node

	Case	Metastasis	Cervical
T1a	142	61	32
	12.83%	42.96%	22.54%
T1b	350	239	132
	31.62%	68.29%	37.71%
T1c	369	309	210
	33.33%	83.74%	56.91%
T2	231	203	161
	20.87%	87.88%	69.69%
T3	15	12	11
	1.35%	80%	73.33%
Collection	1107	824	546
		74.44%	49.32%

25.56% of patients had negative lymph nodes in the central and neck regions, and 25.11% of patients had only central lymph node metastasis, that is, 50.67% of patients had cervical lymph nodes that were safe. The proportion of patients with metastasis in both the central area and the neck area was 42.55%, of which 39.49% were from T1c patients. In all patients with positive lymph nodes in the central area, 62.88% of the patients had cervical metastasis. Only 6.78% of patients had jump metastasis, that is, the central lymph nodes were negative, and cervical lymph node metastasis occurred, of which T1c patients accounted for 30.67%, and T1c and above patients accounted for 78.55% (Table 8).

**TABLE 8:** The metastasis of central and lateral neck node

Cervical	Cases	T1a	T1b	T1c	T2	T3
0–0	283	81	111	61	28	2
	25.56%	28.62%	39.22%	21.55%	9.89%	0.71%
0–1	75	11	18	23	20	3
	6.78%	14.67%	24%	30.67%	26.67%	4%
1–0	278	29	107	98	42	2
	25.11%	10.43%	38.49%	35.25%	15.11%	0.72%
1–1	471	21	114	186	141	8
	42.55%	4.46%	24.2%	39.49%	29.94%	1.69%

Of the patients with T1a, 57.04% did not have metastasis. In T1b, there was no metastasis in 31.71% of lymph nodes, 30.57% had only central lymph node metastasis, and 32.57% had metastasis in central and neck regions. In T1c patients, 50.41% of patients had metastasis. In T2 patients, the metastasis rate was 61.04%, and in T3 patients, it was as high as 53.3% (Table 9).

**TABLE 9:** The diameter and metastasis of cervical lymph node

Cervical	Cases	0–0	0–1	1–0	1–1
T1a	142	81	11	29	21
		57.04%	7.75%	20.42%	14.79%
T1b	350	111	18	107	114
		31.71%	5.14%	30.57%	32.57%
T1c	369	61	23	98	186
		16.53%	6.23%	26.56%	50.41%
T2	231	28	20	42	141
		12.12%	8.66%	18.18%	61.04%
T3	15	2	3	2	8
		13.33%	20%	13.33%	53.33%

**FIGURE 2:**
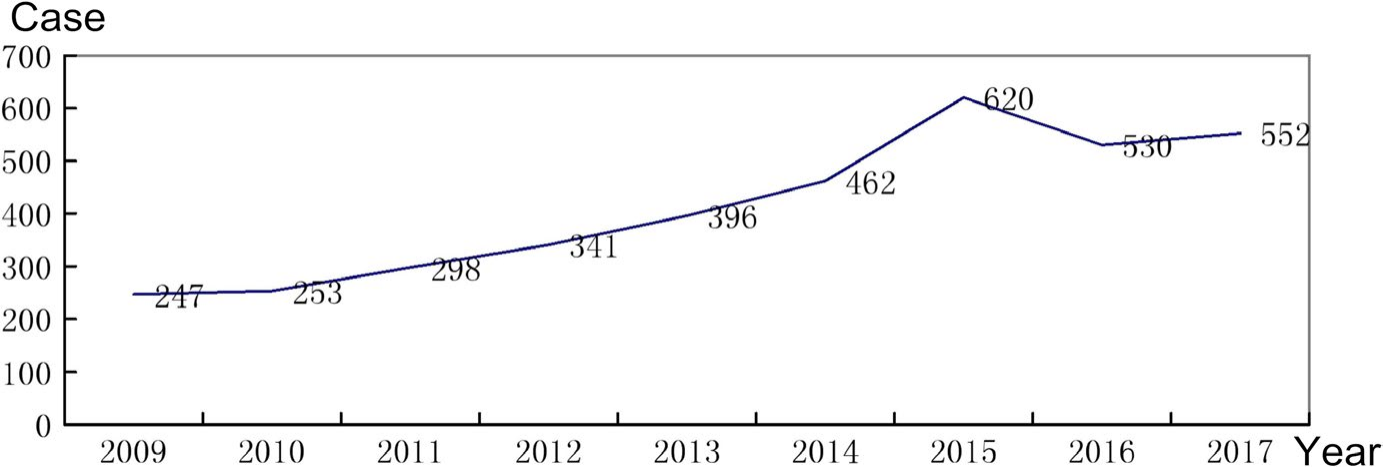
Numbers of cases of thyroid cancer treated at our hospital in the past 9 years

In addition, we enumerated the number of cases of surgery for thyroid cancer at our hospital from 2009 to 2018, and the results demonstrate that the number of cases generally rose annually by 4.72%–18.16% (Figure 2). In the Information Age, people can get the medical information they need more conveniently. With people's increasingly rich material and cultural life, people pay more and more attention to personal physical health, thus more people will consciously take the initiative to have a physical examination. At present, in the era of medical technology innovation, the medical level of clinical, pathological, and imaging doctors has been constantly improved, and the popularity of high‐resolution technical equipment and more accurate diagnosis technology has increased the diagnosis rate of thyroid cancer.

## 4 DISCUSSION

According to the results of the SEER database of the National Cancer Center of the United States, the incidence of both PTC <1.0 cm and thyroid cancers 1.0–4.0 cm increased from 1980 to 2010, especially among highly educated groups.^7^ Statistical data from the United States and South Korea show an increase in the incidence of thyroid cancer.^8^ Further, the results of surveys in Denmark, Finland, Israel, Japan, Spain, and Switzerland also demonstrate increases in thyroid cancer incidence, which is mainly PTC, with significant sex differences in the relative extent of the rise. This phenomenon cannot be fully explained by improvements in the sensitivity of testing methods, nor increased awareness among doctors and patients of screening.^9^ Scholars from other countries have actively researched the risk factors associated with thyroid cancer, and found that autoimmune disorders, ionizing radiation, iodine intake, estrogen, environmental endocrine disruptors, negative psychosocial factors, and heredity may contribute to increases in thyroid cancer incidence.^10^, ^11^

From 1984 to 2010, the incidence of differentiated thyroid carcinoma in the United States increased, with tumors of diameter ≤0.5 rising by 5.09%, while those of 0.5–0.99 cm in diameter increased by 8.45%, average diameter tumors (1.0–1.99 cm) by 3.42%, and large tumors ≥2.0 cm by 2.96%. These data suggest that most papillary thyroid microcarcinoma (PTMC) will develop into tumors with diameter >1 cm, but over varying periods of time. In particular, the possibility of dedifferentiation increases with prolonged tumor‐bearing time and patient age.^12^, ^13^ It is generally recognized that, over time, the degree of malignancy and dedifferentiation of PTC tumors increase; however, when the level of malignancy changes, what causes changes in the tumor genome has not been fully or satisfactorily explained.^14^

As available therapies for thyroid cancer are effective, there is little drive for early diagnosis and surgical treatment, which is counterintuitive and violates the basic principles of pursuing early detection, with the aim of early diagnosis and treatment of malignant tumors. In addition, the psychological pressure on patients of the uncertainty of following up while waiting for the diagnosis of malignancies to change before taking action is undoubtedly considerable. Cancer does not have specific Chinese characteristics. In 2014, the Memorial Sloan‐Kettering Cancer Center in the United States launched a “wait‐and‐see” program for patients with thyroid cancer.^15^ Patients diagnosed with microcarcinoma of the thyroid could choose not to have it removed for a while, but rather to have it checked regularly; however, the vast majority of patients with PTMC did not hesitate to choose surgical treatment. Further, some patients who chose the observation option eventually asked for surgery, and doctors feared being sued for missing the optimal treatment time. Early treatment is not only effective, it is also associated with reduced risk and fewer complications.^16^ Once lymphatic metastases, or distant lung and bone metastases, occur, the cost of medical care often increases considerably, which can even endanger the life of the patient.

According to our clinical and pathological data, as the diameter of the mass increases, the proportion of lymph node metastasis increases, from 44.68% to 77.53%. The proportion of micropapillary carcinoma ≤1 was 47.10%, and the rate of lymph node metastasis was 46.92%, which did not mean that the risk of microcarcinoma was low. Lymph node metastasis is also an indicator of malignancy. With the passage of time, the tumor size, the proportion, and the number of lymph node metastasis will further increase, leading to disease progression, the difficulty and risk of surgery, and the risk of recurrence and metastasis gradually increased. When the mass rises above 2 cm, the lymph node metastasis ratio rises to 77.53%, indicating that thyroid papillary carcinoma is progressing, and the malignant degree and the disdifferentiation increase, although there is no way to evaluate and predict it.

Compared with the National Comprehensive Cancer Network (NCCN) and the American Thyroid Association (ATA) guidelines for the diagnosis and treatment of differentiated thyroid cancer, Chinese guidelines for patients undergoing thyroid surgery to remove all indications do not include any specific age‐based requirements. The NCCN guidelines recommend that patients with PTC aged <15 or >45 years should undergo total thyroidectomy. While the ATA guidelines recommend total thyroidectomy for all patients with PTC with tumor diameters >1 cm, unlike Chinese guidelines. Further, total thyroidectomy is also recommended for all patients with thyroid nodules in the contralateral gland and for patients with PTC with tumor diameters 1.0–1.5 cm, aged >45 years. In addition, Chinese guidelines indicate that all patients with differentiated thyroid cancer should receive central region lymph node dissection, as this provides effective protection from recurrent laryngeal nerve and parathyroid gland involvement. The NCCN and ATA guidelines recommend no dissection of the central lymph node without involvement, except for abnormal palpation or clear metastasis diagnosed by puncture biopsy. Total thyroidectomy can have advantages for patients, including: (1) single treatment of multiple lesions, particularly bilateral cancer lesions; (2) facilitates postoperative monitoring of tumor recurrence and metastasis; (3) beneficial for postoperative I^131^ treatment; (4) reduces the recurrence rate of tumors and the probability of reoperation, which avoids increasing the incidence of serious complications due to reoperation; (5) accurate assessment of postoperative staging and risk stratification of patients; (6) prevention of recurrence of thyroid cancer from development into poorly differentiated thyroid cancer.

The bilateral tumor metastasis rate is 65.27%, the recurrent cancer metastasis rate is 72.21%, the tumor breakthrough membrane metastasis rate is 67.08%, and the metastasis rate is higher than the average metastasis rate. The analysis is statistically significant. When combined with high‐risk factors, such as bilateral lesions, multiple lesions, and tumor rupture membranes, the lymph node metastasis rate will increase significantly.^17^ It is recommended that we conduct more comprehensive preoperative clinical assessments to facilitate us to obtain more comprehensive patient disease information. Thyroidectomy and central lymph node dissection may be more reasonable, thorough, and safe procedures for clinicians to consider. At present, some preoperative examinations used in clinical practice cannot reveal suspected cancer lesions in the contralateral glandular lobe. Total thyroidectomy completely removes any such lesions and glands with potential lesions, thereby avoiding the risk of undetected cancer lesions, reducing the recurrence rate, and improving patient prognosis.^18^, ^19^ In patients with PTC, thyroid globulin levels can be assessed postoperatively to determine whether there is residual tumor or recurrence. For patients with all thyroid tissue removed, there should be no thyroid globulin present in the body; hence, if thyroid globulin is detected in the serum, it generally indicates that some lesion remains or has relapsed, which is also an important prognostic indicator.

Total thyroidectomy increases the incidence of recurrent laryngeal nerve and parathyroid gland injury, and any complications will have a serious impact on the mental health and quality of life of patients postoperatively. Therefore, surgeons must receive strict guidance and training to reduce or avoid surgical complications. Doctors undergoing training who may conduct lateral + isthmus + contralateral subtotal thyroidectomy should ensure that very small amounts of non‐tumor thyroid tissue are left on the contralateral glandular lobe only to avoid recurrent disease of the laryngeal nerve and around the parathyroid gland. Simultaneously, in combination with the application of nanocarbon and nerve monitoring during surgery, to avoid bilateral recurrent laryngeal nerve or parathyroid gland damage, residual thyroid tissue can be dealt with using radioactive I^131^ therapy following surgery.

The cervical lymph node metastasis rate was also positively correlated with the tumor diameter. In all tumors, 49.32% of patients had cervical regional metastasis, of which 69.40% were T1c and above tumor patients. Of the patients with T1c, 76.97% had central lymph node metastasis, of which 65.50% had cervical lymph node metastasis at the same time. Although only 6.78% of patients had jump metastasis, T1c patients accounted for 30.67%, and T1c and above patients accounted for 78.55%. It is suggested that when we treat T1c patients, we should fully evaluate the preoperative evaluation. As far as possible, the original lesion and metastatic lesion should be cleaned together during the initial operation to avoid reoperation. The surgical method can consider cervical lymph node clearance. Thyroid cancer has a good prognosis and long survival. Therefore, we should try our best to protect the function of the tumor while improving the quality of life. It is recommended to keep the sternocleidomastoid, the internal jugular vein, and the accessory nerve, and try to keep the cervical plexus neurocutaneous branch. At present, the modified radical neck dissection (MRND) is the most commonly used procedure for both thorough surgery and preservation of the body function. Lymph node metastasis of thyroid papillary carcinoma is common to the ipsilateral side, and it is transferred along the lymphatic drainage path. The lymphatic metastasis is usually first to the paratracheal lymph node, and then drained to the jugular vein lymph node (level Ⅱ–Ⅳ) and the posterior cervical lymph nodes (level Ⅴ), or down the trachea to the upper mediastinum. The most common site in the sixth district, followed by the neck Ⅲ, Ⅳ, Ⅱ, and Ⅴ levels, and the lymph node metastasis of PTC is mainly metastasis.

At present, surgical treatment of cervical lymph node dissection is recommended for PTC with cervical lymph node metastasis. Prophylactic dissection is not recommended. The occult metastasis of cervical lymph nodes does not reduce the survival rate of patients, so prophylactic cervical lymph node dissection is not recommended in patients with cN0.

For patients with cN1, lymph node metastasis is an independent risk factor for the survival of patients with >45‐year‐old PTC.^20^, ^21^ Levels Ⅱ–Ⅳ are the main area of thyroid lymphatic drainage, and levels Ⅲ and Ⅳ are the most common lymph node metastasis. Domestic experts agree that levels Ⅱa, Ⅲ, Ⅳ, Ⅴb are the standard range of cervical lymph node dissection.^22^ It is recommended that patients with cervical lymph node metastasis confirmed by preoperative evaluation or intraoperative frozen pathological examination receive cervical lymph node dissection, but preventive cervical lymph node dissection is not recommended.^23^ Reoperation will increase the complications of surgery, and it will lead to recurrence of disease after thyroid cancer surgery, and even affect survival. At the same time, as the number of operations increases, the degree of tumor dedifferentiation will increase, and the difficulty and risk of surgery will increase. It is impossible to completely remove the surgery again, so that the patient who can be cured can lose the opportunity to cure.^24^, ^25^

Thyroidectomy can lead to complications, primarily parathyroid injury (3%–5%) and recurrent laryngeal nerve injury (1%–5%). It is accepted that patients who undergo total or partial thyroidectomy must use thyroxine replacement, or even suppression, therapy for the rest of their lives.^26^, ^27^ Data from our hospital related to hypocalcemia, hoarseness, and sensory dysfunction of the shoulder after thyroid surgery currently undergoing statistical analysis and will be presented in future reports. The pathological characteristics of PTC mean that analysis of the influence of surgical approach on survival and prognosis will be a long‐term project, and we will continue to analyze follow‐up data for these patients. Only a small number of patients were found to have BRAF gene mutations in our study, as there is no comprehensive promotion of genetic testing in our hospital, hence not all patients were evaluated.

According to the CTA, endocrine inhibition therapy is essential, regardless of surgical approach. Hence, long‐term endocrine suppression therapy was required for all patients with PTC, including those who underwent lobectomy + central lymph node dissection. Radioactive I^131^ therapy is also an important part of comprehensive treatment of thyroid cancer.^28^, ^29^ All patients with lymph node metastasis, whether in the central or cervical region, were treated with I^131^ administered at doses of 80–130 mCi, according to risk stratification assessment, with additional rounds of treatment if needed, based on the results of follow‐up observation.

The method of treatment of thyroid cancer is influenced not only by surgeons, but also by nuclear medicine, imaging, and other clinicians, and even by health insurance companies. Careful preoperative staging and risk stratification of thyroid carcinoma result in better outcomes for patients with thyroid carcinoma. This is because such an approach facilitates more accurate surgery, and individualized treatment, as well as allowing collection of data on national trends in this disease. The authors consider that surgeons should fully embrace the principles of tumor prevention and treatment, and be mindful that thyroid cancer is a malignant tumor; hence, the scope of treatment should not be reduced unnecessarily, to avoid the need for reoperation several years later. Therefore, it is necessary to assess patients, according to current scientific knowledge, to make a comprehensive judgment of the safety of surgery, the willingness of the patient, and medical resources available, together with the national considerations and regional factors in China, and the degree of acceptance of the operation and possible secondary surgery by the patient. Together, this information can inform implementation of rational diagnosis and treatment plans.

## 5 CONCLUSIONS

The data from our department inform several important points. (1) the incidence of thyroid cancer cases in our hospital generally rose annually during 2009–2018 from a minimum of 247 to a maximum of 600 (increase rates from 4.72% to 18.16%); hence, thyroid cancer has become a common malignancy threatening the physical and mental health of patients and representing a dark psychological shadow and economic burden on both individuals and society. (2) PTC, even PTMC, has a high rate of local metastasis (57.23% and 48.97%, respectively) as, although it is an inert tumor after recovery, high invasiveness can occur on evolution of the tumor genome, indicating that it cannot be considered to exhibit a low degree of malignancy, suggesting that we should give more active and thorough treatment. (3) When there were complications, such as bilateral lesions, multiple lesions, and tumor breakthrough membranes, tumors were highly aggressive, suggesting that priority should be given to total central thyroidectomy lymph node dissection. Postoperative complications and long‐term follow‐up data will be reported in the future. (4) There is no need to prevent cervical lymph node clearance, but when the mass reaches T1c, the risk of central area, neck side area, and jump type metastasis is obviously high. It is recommended to give cervical side lymph node clearance, so that it is more complete and safer.

## CONFLICT OF INTEREST

The authors declare that they have no conflict of interest.

## ETHICS APPROVAL AND CONSENT TO PARTICIPATE

All medical records are legal and reasonable. The study was approved and supervised by the ethics committee of Liaocheng People's Hospital, affiliated to Shandong First Medical University.

## CONSENT FOR PUBLICATION

Written informed consent for publication was obtained from all participants.

## Data Availability

We confirm that we will share the data underlying the findings reported in this manuscripts and allow researchers to verify the results presented, replicate the analysis, and conduct secondary analyses.
